# Clinical Characteristics of Macrolide-Refractory *Mycoplasma pneumoniae* Pneumonia in Korean Children: A Multicenter Retrospective Study

**DOI:** 10.3390/jcm11020306

**Published:** 2022-01-08

**Authors:** Yun Jung Choi, Eun Hee Chung, Eun Lee, Chul-Hong Kim, Yong Ju Lee, Hyo-Bin Kim, Bong-Seong Kim, Hyung Young Kim, Yoojung Cho, Ju-Hee Seo, In Suk Sol, Myongsoon Sung, Dae Jin Song, Young Min Ahn, Hea Lin Oh, Jinho Yu, Sungsu Jung, Kyung Suk Lee, Ju Suk Lee, Gwang Cheon Jang, Yoon-Young Jang, Hai Lee Chung, Sung-Min Choi, Man Yong Han, Jung Yeon Shim, Jin Tack Kim, Chang-Keun Kim, Hyeon-Jong Yang, Dong In Suh

**Affiliations:** 1 Hospital Medicine Center, Seoul National University Hospital, Seoul KS013, Korea; flubber224@gmail.com; 2 Department of Pediatrics, Seoul National University Hospital, Seoul KS013, Korea; 3 Department of Pediatrics, Chungnam National University Hospital, Chungnam National University School of Medicine, Daejeon KS015, Korea; ehchung@cnu.ac.kr; 4 Department of Pediatrics, Chonnam National University Hospital, Chonnam National University Medical School, Gwangju KS018, Korea; unelee@daum.net; 5 Department of Pediatrics, Samsung Changwon Hospital, Sungkyunkwan University School of Medicine, Changwon KS011, Korea; chkimmd@hanmail.net (C.-H.K.); ljs8952194@naver.com (J.S.L.); 6 Department of Pediatrics, Yongin Severance Hospital, Yongin KS009, Korea; LEEYONGJU@yuhs.ac; 7 Asthma and Allergy Center, Department of Pediatrics, Inje University Sanggye Paik Hospital, Seoul KS013, Korea; hbkim1126@naver.com (H.-B.K.); kimck@paik.ac.kr (C.-K.K.); 8 Department of Pediatrics, Ulsan University Gangneung Asan Hospital, Gangneung KS007, Korea; bskped@hanmail.net; 9 Department of Pediatrics, Pusan National University Children’s Hospital, Yangsan KS011, Korea; hawmok77@hanmail.net (H.Y.K.); jung5984@daum.net (S.J.); 10 SCH Biomedical Informatics Research Unit, Soonchunhyang University Seoul Hospital, Seoul KS013, Korea; cyoojung1019@gmail.com; 11 Department of Pediatrics, Dankook University Hospital, Dankook University Medical School, Cheonan KS002, Korea; jhseo@dkuh.co.kr; 12 Department of Pediatrics, Kangbuk Samsung Hospital, Sungkyunkwan University School of Medicine, Seoul KS013, Korea; issolkk0312@gmail.com (I.S.S.); jy7.shim@samsung.com (J.Y.S.); 13 Department of Pediatrics, Soonchunhyang University Gumi Hospital, Gumi KS010, Korea; myong47@hanmail.net; 14 Department of Pediatrics, Korea University Guro Hospital, Korea University College of Medicine, Seoul KS013, Korea; djsong506@korea.ac.kr; 15 Department of Pediatrics, Eulji General Hospital, Eulju University, Seoul KS013, Korea; ymahn964@naver.com; 16 Department of Pediatrics, Korea Cancer Center Hospital, Seoul KS013, Korea; drhealin@gmail.com; 17 Department of Pediatrics, Asan Medical Center, University of Ulsan College of Medicine, Seoul KS013, Korea; jyu3922@gmail.com; 18 Department of Pediatrics, Hanyang University Guri Hospital, Hanyang University College of Medicine, Guri KS013, Korea; lksallergy@gmail.com; 19 Department of Pediatrics, National Health Insurance Service, Ilsan Hospital, Goyang KS007, Korea; spajang@naver.com; 20 Department of Pediatrics, Catholic University of Daegu School of Medicine, Daegu KS002, Korea; yyjang0117@gmail.com (Y.-Y.J.); hlchung@cu.ac.kr (H.L.C.); 21 Department of Pediatrics, Dongguk University Gyeongju Hospital, Gyeongju KS010, Korea; csm21@dongguk.ac.kr; 22 Department of Pediatrics, CHA Bundang Medical Center, CHA University School of Medicine, Seongnam KS009, Korea; drmesh@gmail.com; 23 Department of Pediatrics, College of Medicine, The Catholic University of Korea, Seoul KS013, Korea; jintackk@catholic.ac.kr; 24 Department of Pediatrics, Soonchunhyang University Seoul Hospital, Soonchunhyang University College of Medicine, Seoul KS013, Korea

**Keywords:** *Mycoplasma pneumoniae* pneumonia, macrolide refractory *Mycoplasma pneumoniae* pneumonia, children

## Abstract

*Mycoplasma pneumoniae* is a major causative pathogen of community-acquired pneumonia in children, and the treatment of choice is macrolides. There is an increasing trend in reports of refractory clinical responses despite macrolide treatment due to the emergence of macrolide-resistant *M. pneumoniae*. Early discrimination of macrolide-refractory *M. pneumoniae* pneumonia (MrMP) from macrolide-sensitive *M. pneumoniae* pneumonia (MSMP) is vital; however, testing for macrolide susceptibility at the time of admission is not feasible. This study aimed to identify the characteristics of MrMP in Korean children, in comparison with those of MSMP. In this multicenter study, board-certified pediatric pulmonologists at 22 tertiary hospitals reviewed the medical records from 2010 to 2015 of 5294 children who were hospitalized with *M. pneumoniae* pneumonia and administered macrolides as the initial treatment. One-way analysis of variance and the Kruskal-Wallis test were used to compare differences between groups. Of 5294 patients (mean age, 5.6 years) included in this analysis, 240 (4.5%), 925 (17.5%), and 4129 (78.0%) had MrMP, macrolide-less effective *M. pneumoniae* pneumonia, and MSMP, respectively. Compared with the MSMP group, the MrMP group had a longer fever duration, overall (13.0 days) and after macrolide use (8.0 days). A higher proportion of MrMP patients had respiratory distress, pleural effusion, and lobar pneumonia. The mean aspartate aminotransferase, alanine aminotransferase, lactate dehydrogenase, and C-reactive protein levels were the highest in the MrMP group, along with higher incidences of extrapulmonary manifestations and atelectasis (during and post infection). Pre-existing conditions were present in 17.4% (*n* = 725/4159) of patients, with asthma being the most common (*n* = 334/4811, 6.9%). This study verified that MrMP patients show more severe initial radiographic findings and clinical courses than MSMP patients. MrMP should be promptly managed by agents other than macrolides.

## 1. Introduction

*Mycoplasma pneumoniae* is one of the major causative pathogens of community-acquired pneumonia in children. This pneumonia follows a cyclic epidemic pattern. Although *M. pneumoniae* infections with mild symptoms can be resolved without treatment, these infections can sometimes progress to fulminant or necrotizing pneumonia with respiratory distress syndrome. Macrolides have traditionally been considered the treatment of choice for *M. pneumoniae* pneumonia (MP) in children [1]. In the past decade, there has been an increasing trend in macrolide-refractory *M. pneumoniae* pneumonia (MrMP), which does not resolve despite macrolide treatment [2,3,4].

Refractoriness to macrolide treatment in MP is largely thought to be associated with the emergence of macrolide-resistant *M. pneumoniae*. Mutations in 23S ribosomal RNA (rRNA) are key features of macrolide resistance in *M. pneumoniae* [5]. To confirm macrolide resistance, either a polymerase chain reaction (PCR) for 23S rRNA mutations or a minimum inhibitory concentration measurement using *M. pneumoniae* cultures is required. PCR assays to identify point-mutations in 23S rRNA are currently commercially unavailable, and it takes at least 2 weeks to obtain culture results [5,6]. For these reasons, it is practically impossible to use information on macrolide resistance in clinical practice. Administering effective antibiotics to pneumonia patients is critical. Among patients with MP, those with macrolide-sensitive *M. pneumoniae* (MSMP) show considerable clinical improvement shortly after macrolide administration [7,8]. Therefore, in the early period of MP, predicting responsiveness to macrolide treatment as well as the macrolide susceptibility of *M. pneumoniae* is of greater significance. Currently, no measures are available.

From a clinical point of view, the response to antibiotics is not affected by antibiotic susceptibility alone. When it comes to MP, which has a self-limiting natural course, other factors such as host condition and the initial disease burden may have a greater impact on the clinical treatment response [5]. It is difficult to predict the treatment response on the day of admission to the hospital. In recent years, there has been increasing interest in differentiating MrMP from MSMP and comparing their clinical features. However, current studies on this topic have been limited to single-center or small-scale studies [4,9,10,11].

Therefore, this study aimed to assess the clinical characteristics of MrMP in Korean pediatric patients and to determine the differences between MrMP and MSMP through a multicenter, retrospective study.

## 2. Materials and Methods

For confidentiality, patient identifiers were not entered into the database used for the study. This study was approved by the Institutional Review Board (IRB) of all institutions related to the study, and the requirement for consent was waived. The list and IRB approval numbers of all the institution related with this study are described in Supplementary file S1. All the authors, as the board-certified specialists in pediatric pulmonology in Korea, collected data and registered it on the internet-based Clinical Research and Trial management system (iCReaT) (assigned No. C170017) that is built and operated by the National Institutes of Health (NIH) in Korea.

The present study retrospectively reviewed data of pediatric patients with community-acquired pneumonia between 1 January 2010, and 31 December 2015. The data were collected in cooperation with tertiary medical institutions under the Pneumonia and Respiratory Disease Study Group of the Korean Academy of Pediatric Allergy and Respiratory Disease [12]. The inclusion criteria for patients in this study were as follows: (i) age < 18 years; (ii) diagnosis of MP; (iii) hospitalization for treatment; and (iv) macrolide administration as the initial treatment. Most of the clinical information and chest radiographs reported in this study were reviewed by pediatric pulmonary specialists. Furthermore, data on the following clinical features were extracted using claim codes, including the use of prescribed oxygen, intensive care unit (ICU) admission, pleural puncture, and chest tube insertion. Laboratory findings during the initial period of hospitalization were collected through computerized data extraction from each hospital.

### 2.1. Study Subjects

**Figure 1:**
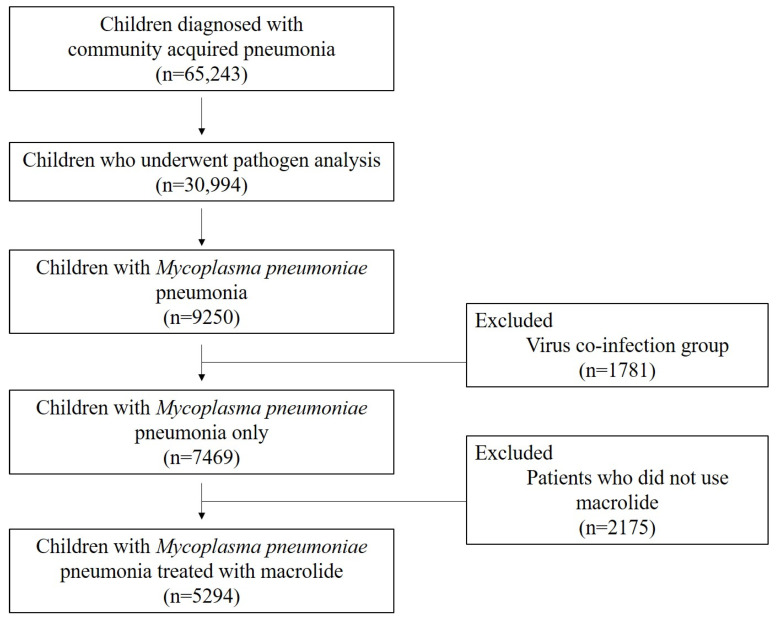
Inclusion Criteria.

Over a 6-year period, data from a total of 65,243 pediatric patients with community-acquired pneumonia under the age of 18 years were collected. Of these, 30,994 patients who underwent pathogen analysis to determine the cause of the disease and 9250 who were diagnosed with MP were selected. After excluding 1781 patients with confirmed virus coinfection, of the remaining 7469 patients, 5294 patients with a history of macrolide treatment were included in the final analysis (Figure 1, Table S1). During the data collection process, each institution verified the eligible population through the following three-step approach: first, claim code data were screened; second, the list of subjects who underwent mycoplasma testing (either PCR analysis or the measurement of the anti-mycoplasma antibody titer [IgM/IgG]) was obtained; and third, board-certified pediatric pulmonologists from each institution reviewed their own institutional medical records regarding the relevance of MP. Subjects were included only when they had positive results for serologic tests (seroconversion of the specific IgM against *M. pneumoniae* or a four-fold or greater increase in IgM or IgG (or both) antibody titers between the acute and convalescent stages) as well as positive results on PCR or real-time PCR for *M. pneumoniae* using nasopharyngeal aspiration or sputum samples [4,11,12,13]. The population was divided into three groups according to the fever duration after macrolide administration, used as a marker for clinical response to treatment [4,12,14]: MrMP (for those who had fever for ≥7 days after macrolide administration), macrolide-less effective MP (MLMP; for those with fever for ≥3 days but <7 days), and MSMP (for those whose fever subsided within 3 days).

We reviewed the patients’ age, sex, change in clinical symptoms during hospitalization, hospital stay, and fever duration before and after macrolide use. The presence and severity of respiratory distress were determined by the pediatric pulmonologists of each institution through chart review for data on signs of abnormal breathing, including chest retraction (suprasternal, intercostal, or subcostal), grunting, nasal flaring, and paradoxical chest wall movement. Respiratory distress was categorized as mild, moderate, or severe based on the Japanese Guidelines for the Management of Respiratory Infectious Diseases in Children 2007, with a focus on pneumonia [15]. We also examined whether the patient received oxygen, whether they were in ICU, or were treated with mechanical ventilation. Information on patients’ underlying diseases and long-term or short-term complications associated with MP were also collected.

### 2.2. Laboratory Tests and Image Study

The laboratory results were collected on the day of hospitalization, including those of the neutrophil count, lymphocyte ratio, eosinophil ratio, platelet count, C-reactive protein (CRP) level, erythrocyte sedimentation rate (ESR), aspartate aminotransferase (AST) level, alanine aminotransferase (ALT) level, and lactate dehydrogenase (LDH) level.

We collected chest radiograph findings during hospitalization. Chest radiographs were classified to show bronchopneumonia or lobar pneumonia with or without pleural effusion and atelectasis. We also checked the list of patients who needed invasive procedures.

### 2.3. Statistical Analyses

Categorical variables were summarized as frequency (%), and serial data were summarized as the mean and standard deviation when the assumption of normal distribution was satisfied. Serial data were summarized as the median and interquartile range when the assumption of normal distribution was not satisfied. One-way analysis of variance was used to compare differences between three or more consecutive variables showing a normal distribution; the Kruskal-Wallis test, to compare differences between median data not showing a normal distribution; and Dunn multiple comparisons test, to perform post-validation analysis. We performed multivariable logistic regression using statistical significant variables and selected on the basis of the lowest Akaike information criterion (AIC). Statistical analysis was performed using the SAS Enterprise Guide software (version 6.1) and R 3.5.1 version (R Foundation for Statistical Computing, Vienna, Austria). *p* values less than 0.05 were considered statistically significant.

## 3. Results

### 3.1. Subject’s Demographics and Comparison of the Patient’s Clinical Characteristics

A total of 5294 patients who were hospitalized with a diagnosis of mycoplasma pneumonia between 1 January 2010 and 31 December 2015, met the inclusion criteria. Patient demographics are shown in Table 1. The mean age was 5.6 years, and 49.0% of the patients were male. There were 240 (4.5%), 925 (17.5%), and 4129 (78.0%) patients with MrMP, MLMP, and MSMP, respectively. The mean duration of fever (from the initial onset of fever) in all patients was 6.4 days. Of 5294 patients, 744 (14.1%) had respiratory distress, 231 (4.4%) required oxygen therapy, and 19 (0.4%) required ICU care.

The results of subgroup comparisons of the subjects’ clinical characteristics according to macrolide response are shown in Table 2. The MrMP group had a higher mean age (6.4 years) than the MSMP group (5.4 years) (*p* < 0.01). The median length of hospital stay was 11.0 days in the MrMP group, which was longer than that in the MSMP group (5.0 days) (*p* < 0.01). Among the three groups, the MrMP group had the longest total fever duration (13.0 days) and the longest fever duration after macrolide use (8.0 days) (*p* < 0.01). Higher proportions of patients with respiratory distress (21.7% vs. 13.6%) and moderate or more severe respiratory distress (15.4% [8/52] vs. 8.5% [44/518]) were noted in the MrMP group than in the MSMP group (*p* < 0.01).

Oxygen saturation (measured via pulse oximetry) ≤ 90% or cyanosis was observed more commonly in the MrMP group (6.9%) than in the other two groups. A higher proportion of patients received oxygen therapy in the MrMP group (12.1%) than those in the other two groups (*p* < 0.01). The proportion of ICU admissions was higher in the MrMP group (0.8%, 2/240 patients) than in the MSMP group (0.3%, 13/925 patients).

### 3.2. Laboratory Findings

Laboratory results of the major inflammatory markers at admission and comparisons of these results between the groups are shown in Table 3. The mean white blood cell (WBC) count of 9.4 × 109/L was highest in the MSMP group (mean 9.7 × 109/L) and lowest in the MrMP group (mean 8.1 × 109/L) (*p* < 0.01). The MrMP group had the highest percentage of neutrophils (63.9%) but the lowest percentage of lymphocytes (26.1%). A significantly higher AST level was found in the MrMP group (mean 73.4 IU/L) than in the MLMP (mean 51.2 IU/L) or MSMP group (mean 38.0 IU/L). Mean levels of ALT, LDH, and CRP were higher in the MrMP group than in the MSMP group (*p* < 0.01 for all).

Receiver operating characteristic curves for determining the cut-off levels of AST, ALT, LDH, and CRP for predicting MrMP are shown in Figure S1. At an AST value of 35, the sensitivity and specificity of predicting MrMP were 56.7% and 66.8%, respectively. Meanwhile, the cut-off values for ALT, LDH, and CRP were 18.0 IU/L, 659 IU/L, and 3.34 g/L, respectively. At the cut-off value, LDH showed high specificity (81%) but low sensitivity (44%) (Figure S1).

### 3.3. Chest Imaging Findings

Features on simple chest radiography at admission and the characteristics of each group are shown in Table 4. Bronchial involvement and lobar pneumonia were present in 67.2% (3339/4972) and 45.4% of patients, respectively. In 17.3% (861/4967) of patients, both bronchial involvement and lobar involvement were observed. The proportion of patients with lobar pneumonia in the MrMP group (65.0%) was similar to that in the MLMP group (60.0%) but significantly higher than that in the MSMP group (41.0%) (*p* < 0.01). Meanwhile, in terms of lobar pneumonia alone, all three groups had a high proportion of unilateral lobar involvement (37.4–59.5%), and the two most involved lobes were the right and left lower lobes in all three groups when overlap was allowed. Atelectasis was found in 5.5% (273/4967) of patients overall, with a higher proportion in the MrMP group (10.8%) than in the MLMP (6.4%) and MSMP (4.9%) groups (*p* < 0.01).

The relative distribution by size of pleural effusion, a major pulmonary complication in MP, is shown in Table 5. Pleural effusion was observed in a total of 402 patients (7.7%), and in 0.5% (2/402) of these patients, half or more than half of the thorax was involved. The proportion of those with pleural effusion was significantly higher in the MrMP group (16.7%) than in the MSMP group (5.8%). When pleural effusion was present, a significantly higher proportion of patients with more than a quarter of the thorax involved (3/40, 7.5%) occurred in the MrMP group than in the MLMP (1/125, 0.8%) or MSMP groups (3/235, 1.3%). Consequently, the percentage of patients requiring chest tube insertion was highest in the MrMP group than in the MLMP and MSMP groups (6.7% vs. 1.3% vs. 0.3%, respectively, *p* < 0.01).

### 3.4. Extrapulmonary Manifestations and Postinfectious Sequalae

Different extrapulmonary manifestations in patients with MP and their relative distribution are shown in Table 6. In total, 22.2% (1091/4910) of the patients had one or more extrapulmonary manifestations, and significantly more extrapulmonary manifestations were noted in the MrMP group (41.4%) than in the MSMP group (18.8%). Liver enzyme elevation, which is indicative of hepatobiliary system involvement, was the most common (17.2%, 857/4990), followed by proteinuria (4.2%, 208/4928) and skin and mucosal involvement (4.1%, 216/5294). Comparisons between the groups with respect to organ involvement revealed that liver enzyme elevation was the most common in the MrMP group (32.5%) and least common in the MSMP group (14.1%) (*p* < 0.01). Similar results were observed for proteinuria (*p* < 0.01) and skin and mucosal involvement (*p* < 0.01). Cardiovascular, nervous system, and musculoskeletal system involvement was observed in only a small number of patients (0.4%, 0.4%, and 0.2%, respectively); however, these manifestations were the most common in the MrMP group.

Postinfectious pulmonary sequelae observed after recovery from MP and their relative distribution are shown in Table 7. Atelectasis persisted in a total of 48 patients (1.6%), and bronchiolitis obliterans was found in 22 patients (0.4%). Persistent atelectasis was more common in the MrMP group (3.2%) than in the MSMP group (1.2%). There were no differences in the number of bronchiolitis obliterans cases among the three groups.

### 3.5. Patient’s Pre-Existing Conditions

The comparison of pre-existing conditions in each group is presented in Table S2. A total of 725 patients (17.4%) had pre-existing conditions. The most common pre-existing condition was asthma (334 of 4811, 6.9%), followed by congenital heart disease (56/5224, 1.1%), and neurologic disorders (47/4996, 0.9%). Bronchopulmonary dysplasia, a common risk factor for respiratory disease in children, was found in only 14 patients (0.3%). Among the three groups, the proportion of patients with asthma was the highest in the MSMP group (286/3703, 7.7%). The proportion of patients with hemato-oncological disorders was higher in the MrMP group than in the MSMP group (*p* < 0.01).

### 3.6. Prediction Model

Predictive modeling was performed through logistic regression analysis using statistical significant variables that showed differences in the MrMP group, and the best model with the lowest AIC was selected. The variables used were respiratory distress, oxygen saturation ≤ 90% or cyanosis, oxygen support during hospitalization, oxygen support (days), AST, ALT, CRP, lobar pneumonia at admission, atelectasis at admission, pleural effusion at admission and any extrapulmonary manifestations. LDH was excluded from the analysis due to its high non-response rate. As a result, the combination of respiratory distress, oxygen saturation ≤ 90% or cyanosis, oxygen support during hospitalization, ALT, CRP, lobar pneumonia at admission and any extrapulmonary manifestations was the best model for predicting MrMP. This model showed 0.70 of the area under the curve (AUC), the sensitivity and specificity were 72.9% and 57.0% (Figure S2).

## 4. Discussion

This study reviewed a total of 5294 patients with MP who were administered macrolides as the initial treatment. Of these patients, 78.0% (4129) responded to treatment promptly, whereas 4.5% (240) had fever for ≥1 week despite macrolide treatment. The MrMP group showed clinical characteristics different from those of the MSMP group, including a longer duration of fever after hospitalization and consequently, a longer length of hospital stay. The proportions of patients with respiratory distress during hospitalization and those with moderate to severe respiratory distress were also higher in the MrMP group than in the MSMP group. Chest radiography findings at admission showed that bronchopneumonia was more common in the MSMP group, whereas the proportions of lobar pneumonia and bronchopneumonia were similar in the MrMP group. During treatment, pleural effusion was more commonly observed in the MrMP group than in the MSMP group, with 7.5% of the MrMP group showing involvement of more than one-quarter of the thorax. This is the first large-scale study to perform an extensive review of data on the clinical, radiological, and diagnostic characteristics of patients with MP who showed a refractory response to initial macrolide treatment.

This study was designed to understand the characteristics of MrMP in children more accurately. The authors collected data from nearly 7500 patients with MP hospitalized in 22 tertiary hospitals. Considering that there are only a total of 42 tertiary hospitals in South Korea, this study includes data of the majority (52.4%) of patients admitted to these tertiary hospitals. Moreover, since patients with mild MP are generally treated in primary or secondary hospitals, patients with more severe disease or those who require longer treatment are hospitalized in tertiary hospitals. The results of this study reflect the cultural characteristics of South Korea, where pediatric patients can be hospitalized according to criteria such as poor general condition, even if they are not in critical condition. Nevertheless, the results of this study may be representative of patients with MP including moderate to severe MP in South Korea. Moreover, this study did not merely analyze claim codes or coded data based on the diagnosis. Instead, pediatric pulmonary specialists at each hospital reviewed the medical records, and high-quality data with high validity were collected at the data collection step.

The MrMP group showed a higher proportion of respiratory distress with greater severity than the MSMP group. This result contradicts previous reports that found no difference in the incidence of respiratory distress between the MrMP and MSMP groups [10,16]. While it is well known that dyspnea is an accompanying symptom of MP, the reported prevalence of dyspnea varies widely from 8 to 83% between studies [17,18,19]. This variation may be explained by the small number of subjects (around 100 from a single center) in most previous studies. Furthermore, previous studies did not have enough power to detect significant differences between groups because of their small sample sizes, compared to that of the present multicenter, large-scale study. Although some studies have reported a significant difference in the prevalence of respiratory distress, no study has investigated the distribution of its severity [20]. As we classified patient groups clinically based on the duration of the fever, it is difficult to discuss our findings in association with the effect of macrolide resistance. However, this study is important in that it reported the actual prevalence of respiratory distress and its distribution by severity in a large number of patients with MrMP as well as in the whole MP patient population.

In addition to respiratory distress, other markers of pneumonia severity were worse in the MrMP group than in the MSMP group. The proportion of patients with oxygen desaturation requiring oxygen therapy or ICU care was also higher in the MrMP group. Assuming that that the MrMP group may include a large number of patients with macrolide-resistant *M. pneumoniae* pneumonia and that excessive inflammation is observed in patients with macrolide-resistant *M. pneumoniae* pneumonia [1,21], a slower response to treatment and higher severity are expected in the MrMP group.

Chest radiography at admission showed a greater degree of lobar pneumonia than bronchopneumonia in the MrMP group, and the incidence rate of lobar pneumonia was higher in the MrMP group than in the MSMP group. In addition, the proportion of those with pleural effusion or atelectasis was higher in the MrMP group than in the MSMP group. This is consistent with previous reports [10,22,23] and is in accordance with the results of another study, which showed that pneumonia with effusion or with the involvement of two or more lobes is more common in patients with MrMP than in those with MSMP [4]. However, another study has reported a contrasting result [24], which may be related to the lower statistical power due to the small sample size. In contrast to previous studies, the present study even examined differences in effusion size. We found a higher prevalence of severe effusion (involvement of more than one-quarter of the thorax) in the MrMP group (7.5%) than in the MSMP group (1.3%). The radiologic findings indicating lobar pneumonia and massive pleural effusion in the MrMP group suggest severe illness due to macrolide resistance, a higher *M. pneumoniae* burden, severe host reactions, or other factors related to the refractory response. In patients with MP, forthcoming refractory response to macrolide needs to be taken into consideration if massive pleural effusion or lobar pneumonia is detected on chest radiography during the initial period of hospitalization.

In such situations, alternative options should be actively considered instead of continuing treatment with macrolide. Immunomodulators to control excessive immune responses or stepwise antibiotic therapy using quinolones can be adopted [25,26]. Various studies have reported that treatment with immunomodulators or alternative antibiotics for MrMP has improved the clinical course. Furthermore, the Korean guideline for the treatment of mycoplasma pneumonia in children also recommends this approach; tetracycline or quinolone or a combination of corticosteroids is recommended for severe pneumonia that does not improve within 72 h of macrolide administration [27]. In the future, a large-scale study on second-line treatment against MP, including the relationship between macrolide-resistant MP and MrMP, is warranted.

Extrapulmonary manifestations are common in MP, and some extrapulmonary manifestations can cause more serious medical problems than pneumonia [28]. These problems include encephalitis, Stevens–Johnson syndrome, and myocarditis, which can be observed in up to 25% of patients [1,29]. It is not yet understood whether serious extrapulmonary manifestations are more common in MrMP than in MSMP. In the present study, higher rates of liver function abnormalities were found in MrMP than in MSMP. Furthermore, skin and mucosal manifestations and proteinuria were more common in MrMP. However, contrary to previous reports, central nervous system involvement was rare [30,31,32]. Owing to the differences in prevalence according to region or race, follow-up reports are needed to assess and compare the prevalence and characteristics of extrapulmonary manifestations in other regions and other races.

In the present study, asthma was the most prevalent pre-existing condition in patients with MP. It is well known that mycoplasma infection is associated with and is a risk factor for asthma [33,34,35]. While it is known that patients with asthma are generally susceptible to pneumonia, large-scale studies on whether asthma is a risk factor for mycoplasma pneumonia are rare. Moreover, even fewer studies have examined whether the risk of pneumonia changes in patients with asthma depending on macrolide responsiveness. However, one study reported that the proportion of patients who had asthma or atopic sensitization was higher in the MrMP group than in the MSMP group [24], which is contrary to the results of the present study. Further studies are warranted to resolve these conflicting issues. In contrast, pre-existing conditions observed at higher rates in the MrMP group than in the MSMP group included neurologic and hemato-oncologic disorders. However, the number of patients with these pre-existing conditions was too small, and it was not possible to conclude that patients with these conditions were at higher risk of MrMP.

Prediction of MrMP at the early stages of MP is critically important. In this study, several serologic markers were tested to assess whether they could predict MrMP. As in previous reports [4,10], the leukocyte count was within the normal range in all three groups, but AST, ALT, LDH, and CRP were significantly higher in the MrMP group. Although significant differences in inflammatory markers were observed between the two groups, these markers were not found to be adequate for predicting MrMP. As the combined sensitivity and specificity of the variables are quite low, their clinical usefulness is questionable. Even LDH, which used to be a promising candidate [11,36,37], showed high specificity but low sensitivity and discriminating power. This may be due to the large overlap of serum inflammatory markers between groups or the inherent characteristics that these markers are nonspecific and reflect the level of whole-body inflammation. Other inflammatory cytokines that were not examined in the present study need to be evaluated in terms of whether they perform better in this prediction. Interleukin (IL)-8, IL-18, and tumor necrosis factor (TNF)-alpha, independently or in combination with existing biomarkers, have been tested regarding their predictability [12,24,37,38]. It was attempted to find an appropriate predictive model by adding clinical variables to the inflammatory markers. However, this model was expected to be difficult to use clinically because the sensitivity and specificity were statistically low.

In this study, the authors defined patients with mycoplasma pneumonia that fall in the gray zone between MrMP and MSMP as the MLMP group. The MLMP group was defined as patients showing a fever that lasted for ≥3 days but <7 days; therefore, this group consisted of patients who eventually responded to macrolide within 7 days and were included in the MSMP or general mycoplasma pneumonia group in previous studies. These patients are different from patients with MSMP with respect to radiological findings and extrapulmonary manifestations and different from patients with MrMP with respect to clinical findings; however, no consistent trend was found. MLMP patients may be a heterogeneous population in terms of macrolide sensitivity, extent of involvement, and additional medical measures. Therefore, the MLMP group was excluded from the analysis on group-specific characteristics.

The present study has several limitations. First, this study collected and analyzed the clinical records from multiple centers retrospectively. We have not gathered the treatment details that may affect the disease courses and the case classification. Therefore, there might be an inter-institutional or inter-individual variation regarding the doses and types of initial macrolides and whether to use alternative antibiotics or combined medications such as corticosteroids. However, most researchers that participated in this multi-institutional study are board-certified pediatric pulmonologists that should take biannual update programs and belongs to a same study group. Clinical practices are not so much deviated from the standard ones. For example, macrolides were prescribed and administered in accordance with the general recommended dose within the Ministry of Food and Drug Safety range or the pediatric dosage handbook guidance. Moreover, we limited intergroup comparisons on the data around the admission day. Second, viral coinfection has not been verified in one-third of subjects. We could not fully exclude the issue of misregarding the grave presentation by co-infected viruses, i.e., adenovirus, as the refractory responses to macrolides. Third, in this study, we did not collect information on the types of diagnostic methods for each patient, so it was not possible to classify patients by diagnostic tools, whether only one of PCR and serology tests was performed or both tests were performed. There is a difference in sensitivity and specificity between PCR and serology tests, and a combination of the detection of IgM antibodies and PCR is recommended as a method for detecting mycoplasma infection [39,40]. However, each institution’s board-certified pediatric respiratory specialists reviewed their own institutional medical records for accuracy of diagnosis. They strictly checked whether a patient’s test results met the positive criteria for diagnosis of mycoplasma infection. Finally, the present study did not analyze MPs that were treated in either outpatient-based primary clinics or secondary hospitals. Consequently, it is likely that the characteristics of the MSMP group found in this study may be more severe than those of the actual MSMP group. Nonetheless, the fact that the MSMP group in the present study showed many characteristics that differentiated them from the MrMP group with respect to severity supports the hypothesis that there is a significant difference in disease severity between MSMP and MrMP in actual clinical settings.

## 5. Conclusions

This large-scale, retrospective, multi-institutional study evaluated the characteristics of children with MP based on the clinical response to macrolides. We verified that patients with MrMP present with more severe initial radiographic findings and clinical courses than patients with MSMP. MrMP should be promptly identified, and an alternative treatment strategy must be implemented immediately.

## Figures and Tables

**Table 1:** Subject’s demographics.

Variables	No. (%) or Mean ± SD
Total number of patients	5294
Age, years	5.6 (±3.3)
Male sex	2596 (49.0)
Allergy	714 (13.5)
Bronchopulmonary dysplasia	14 (0.3)
MrMP/MLMP/MSMP	240 (4.5)/925 (17.5)/4129 (78.0)
Total duration of fever (from the initial onset of fever), days	6.4 (±4.2)
Respiratory distress	744 (14.1)
Mild/moderate to severe	668 (12.6)/76 (1.4)
Oxygen support	231 (4.4)
ICU admission	19 (0.4)
Hospital days (days)	6.7 (±4.5)

All data are presented as either the number (%) or mean (±SD). Abbreviations: MrMP, macrolide-refractory *M. pneumoniae* pneumonia; MLMP, macrolide-less effective *M. pneumoniae* pneumonia; MSMP, macrolide-sensitive *M. pneumoniae* pneumonia; ICU, intensive care unit; SD, standard deviation.

**Table 2:** Comparison of clinical characteristic in *Mycoplasma pneumoniae* pneumonia patients.

Category	MrMP		MLMP		MSMP		*p* -Value
Number of Subjects	240	Number of Actual Responses	925	Number of Actual Responses	4129	Number of Actual Responses	
Male sex, *n* (%)	117 (48.8)	240	444 (48.0)	925	2035 (49.3)	4129	0.78
Age (years), mean (SD)	6.4 (3.1)	240	6.1 (3.2)	925	5.4 (3.3)	4129	<0.01 ^†^
Hospital stay (days), median (IQR)	11.0 (8.0–14.0)	240	8.0 (6.0–9.0)	925	5.0 (4.0–7.0)	4129	<0.01 *
Fever duration (days)							
Total, median (IQR)	13.0 (11.0–16.0)	237	9.0 (7.0–11.0)	913	5.0 (3.0–7.0)	3695	<0.01 *
Before admission, median (IQR)	5.0 (3.0–7.0)	237	5.0 (3.0–7.0)	913	4.0 (2.0–7.0)	3696	<0.01 ^†^
After macrolide administration, median (IQR)	8.0 (7.0–9.0)	240	4.0 (3.0–5.0)	925	0.0 (0.0–2.0)	4129	<0.01 *
During hospitalization, median (IQR)	8.0 (6.0–11.0)	240	4.0 (3.0–6.0)	925	1.0 (0–2.0)	4035	<0.01 *
Respiratory rate on admission (/min), median (IQR)	26.0 (24.0–28.0)	240	24.0 (23.0–28.0)	924	24.0 (23.0–28.0)	3814	0.63
Respiratory distress, *n* (%)	52 (21.7)	240	174 (18.9)	922	518 (13.6)	3810	<0.01
Mild, *n* (%)	44 (84.6)		150 (86.2)		474 (91.5)		
Moderate to severe, *n* (%)	8 (15.4)		24 (13.8)		44 (8.5)		
Oxygen saturation ≤ 90% or cyanosis, *n* (%)	16 (6.9)	233	34 (3.8)	897	47 (1.3)	3732	<0.01
Oxygen support during hospitalization, *n* (%)	29 (12.1)	240	52 (5.6)	925	150 (3.6)	4129	<0.01
Oxygen support (days), median (IQR)	4.0 (2.0–6.0)	240	2.0 (1.0–6.0)	925	2.0 (1.0–4.0)	4129	0.01 ^‡^
Admission to the ICU, *n* (%)	2 (0.8)	240	4 (0.4)	925	13 (0.3)	4129	0.1
Mechanical ventilation, *n* (%)	0 (0)	240	4 (0.4)	925	5 (0.1)	4129	

All data are presented as either the number (%), mean (±SD), or median (IQR). For items with missing responses, the number of actual responses was entered in the column on the right. Post-hoc test: *: MrMP > MLMP > MSMP, ^†^: MrMP = MLMP > MSMP, ^‡^ MrMP> MLMP = MSMP Abbreviations: MrMP, macrolide-refractory *M. pneumoniae* pneumonia; MLMP, macrolide-less effective *M. pneumoniae* pneumonia; MSMP, macrolide-sensitive *M. pneumoniae* pneumonia; ICU, intensive care unit; SD, standard deviation; IQR, interquartile range.

**Table 3:** Comparison of serum markers at admission among the clinical response groups.

Categories	Total Population		MrMP		MLMP		MSMP		*p* -Value
Number of Subjects	5294	Number of Actual Responses	240	Number of Actual Responses	925	Number of Actual Responses	4129	Number of Actual Responses	
WBC, 10^9^/L	9.4 (4.7)	5213	8.1 (5.7)	231	8.2 (3.7)	905	9.7 (4.8)	4077	<0.01 ^§^
Neutrophil, %	58.3 (16.9)		63.9 (15.3)		61.3 (16.6)		57.3 (16.9)		
Lymphocyte, %	30.4 (14.1)		26.1 (12.4)		26.7 (11.9)		31.5 (14.4)		
Eosinophil, %	2.8 (3.1)		2.3 (3.0)		2.6 (3.2)		2.9 (31.0)		
Aspartate aminotransferase (AST), IU/L	41.9 (88.4)	5129	73.4 (213.9)	234	51.2 (100.2)	892	38.0 (70.9)	4003	<0.01 *
Alanine aminotransferase, (ALT), IU/L	25.9 (72.6)	4954	54.1 (185.5)	226	32.6 (82.7)	863	22.8 (56.3)	3865	<0.01 *
Lactate dehydrogenase (LDH), IU/L	529.9 (333.7)	2266	746.1 (635.1)	121	627.6 (451.8)	484	485.8 (235)	1661	<0.01 *
C-reactive protein (CRP), g/L	8.1 (19.2)	5018	15 (37.9)	223	11.2 (21.1)	880	7 (16.9)	3915	<0.01 *

All data are presented as the mean (±SD). For items with missing responses, the number of actual responses was entered in the column on the right. Post-hoc test: *: MrMP > MLMP > MSMP, ^§^: MrMP = MLMP < MSMP Abbreviations: MrMP, macrolide-refractory *M. pneumoniae* pneumonia; MLMP, macrolide-less effective *M. pneumoniae* pneumonia; MSMP, macrolide-sensitive *M. pneumoniae* pneumonia; WBC, white blood cell count.

**Table 4:** Initial chest imaging findings in *Mycoplasma pneumoniae* pneumonia patients.

Category	Total Population		MrMP		MLMP		MSMP		*p*-Value
Number of Subjects	5294	Number of Actual Responses	240	Number of Actual Responses	925	Number of Actual Responses	4129	Number of Actual Responses	
Bronchopneumonia	3339 (67.2)	4972	131 (54.6)	240	515 (55.7)	925	2693 (70.7)	3807	<0.01
Lobar pneumonia	2378 (45.4)	5235	156 (65.0)	240	555 (60.0)	925	1667 (41.0)	4070	<0.01
Bilateral	197 (3.8)	5213	12 (5.1)	237	48 (5.2)	921	137 (3.4)	4055	0.02
Unilateral	2159 (41.4)	5215	141 (59.5)	237	503 (54.6)	921	1515 (37.4)	4051	<0.01
Both bronchial and lobar involvement	861 (17.3)	4967	52 (21.7)	240	167 (18.1)	925	642 (16.9)	3802	0.14
Atelectasis	273 (5.5)	4967	26 (10.8)	240	59 (6.4)	923	188 (4.9)	3804	0.01

All data are presented as the number (%) For items with missing responses, the number of actual responses was entered in the column on the right. Abbreviations: MrMP, macrolide-refractory *M. pneumoniae* pneumonia; MLMP, macrolide-less effective *M. pneumoniae* pneumonia; MSMP, macrolide-sensitive *M. pneumoniae* pneumonia.

**Table 5:** Pleural effusion in *Mycoplasma pneumoniae* pneumonia and interventional approach.

Category	Total Population		MrMP		MLMP		MSMP		*p*-Value
Number of Subjects	5294	Number of Actual Responses	240	Number of Actual Responses	925	Number of Actual Responses	4129	Number of Actual Responses	
Pleural effusion	402 (7.7)	5240	40 (16.7)	240	125 (13.5)	925	237 (5.8)	4075	<0.01
<1/4 of the thorax ^‖^	290 (72.1)		30 (75.0)		99 (79.2)		161 (68.5)		<0.01
1/4-1/2 of the thorax ^‖^	5 (1.2)		2 (5.0)		0 (0)		3 (1.3)		
≥1/2 of the thorax ^‖^	2 (0.5)		1 (2.5)		1 (0.8)		0 (0)		
Missing/unknown	105 (26.2)		7 (17.5)		25 (0.2)		73 (31.1)		
Intervention									
Thoracentesis	12 (0.2)	5240	4 (1.7)	240	4 (0.4)	925	4 (0.1)	4129	0.01
Chest tube insertion	39 (0.7)	5240	16 (6.7)	240	12 (1.3)	925	11 (0.3)	4129	<0.01

All data are presented as the number (%). For items with missing responses, the number of actual responses was entered in the column on the right. ^‖^: The ratio was confirmed with the denominator of the patient group with pleural effusion and not the number of total respondents. Abbreviations: MrMP, macrolide-refractory *M. pneumoniae* pneumonia; MLMP, macrolide-less effective *M. pneumoniae* pneumonia; MSMP, macrolide-sensitive *M. pneumoniae* pneumonia.

**Table 6:** Extrapulmonary manifestations.

Category	Total Population		MrMP		MLMP		MSMP		*p*-Value
Number of Subjects	5294	Number of Actual Responses	240	Number of Actual Responses	925	Number of Actual Responses	4129	Number of Actual Responses	
Any manifestation	1091 (22.2)	4910	99 (41.4)	239	284 (31.1)	912	708 (18.8)	3759	<0.01
Digestive system									
Liver enzyme elevation	857 (17.2)	4990	78 (32.5)	240	238 (25.8)	921	541 (14.1)	3829	<0.01
Urinary system									
Proteinuria	208 (4.2)	4928	21 (8.8)	239	51 (5.7)	916	135 (3.6)	3773	<0.01
Skin and mucosa	216 (4.1)	5294	19 (7.9)	240	54 (5.8)		143 (3.5)	4129	<0.01
Rash	212 (4.0)		19 (7.9)		53 (5.7)		140 (3.4)		<0.01
Erythema multiforme	11 (0.2)		3 (1.3)		1 (0.1)		7 (0.2)		0.01
Mucositis	6 (0.1)		0 (0)		1 (0.1)		5 (0.1)		
Cardiovascular system	21 (0.4)	4999	3 (1.3)	240	2 (0.2)	923	16 (0.4)	3836	0.09
Myocarditis	0 (0)		0 (0)		0 (0)		0 (0)		
Kawasaki disease	17 (0.3)		1 (0.4)		2 (0.2)		14 (0.4)		0.08
DIC	4 (0)		2 (0.8)		0 (0)		2 (0.05)		
Nervous system	22 (0.4)	4999	5 (2.1)	240	4 (0.4)	924	13 (0.3)	3835	0.01
Encephalitis	6 (0.1)		1 (0.4)		0 (0)		5 (0.1)		0.22
Meningitis	14 (0.3)		2 (0.8)		4 (0.4)		8 (0.2)		0.10
Peripheral neuropathy	0 (0)		0 (0)		0 (0)		0 (0)		
Musculoskeletal system									
Arthritis	10 (0.2)	5002	3 (1.3)	240	2 (0.2)	924	5 (0.1)	3838	<0.01

All data are presented as the number (%). For items with missing responses, the number of actual responses was entered in the column on the right. Abbreviations: MrMP, macrolide-refractory *M. pneumoniae* pneumonia; MLMP, macrolide-less effective *M. pneumoniae* pneumonia; MSMP, macrolide-sensitive *M. pneumoniae* pneumonia; DIC, disseminated intravascular coagulopathy.

**Table 7:** Postinfectious pulmonary sequelae after *Mycoplasma pneumoniae* pneumonia.

Category	Total Population		MrMP		MLMP		MSMP		*p*-Value
Number of Subjects	5294	Number of Actual Responses	240	Number of Actual Responses	925	Number of Actual Responses	4129	Number of Actual Responses	
Persistent atelectasis	48 (1.6)	2956	5 (3.2)	154	16 (3.2)	506	27 (1.2)	2296	<0.01
Bronchiolitis obliterans	22 (0.4)	5240	1 (0.4)	236	3 (0.3)	911	18 (0.4)	4093	0.90

All data are presented as number (%). For items with missing responses, the number of actual responses was entered in the column on the right. Abbreviations: MrMP, macrolide-refractory *M. pneumoniae* pneumonia; MLMP, macrolide-less effective *M. pneumoniae* pneumonia; MSMP, macrolide-sensitive *M. pneumoniae* pneumonia.

## Data Availability

The data presented in this study are openly available at National Institutes of Health in Korean government (http://nih.go.kr/index.es?sid=a4, accessed on 3 January 2022) in January 2022.

## Supplementary Materials

The following are available online at https://www.mdpi.com/article/10.3390/jcm11020306/s1, Figure S1: Receiver-operating-curves of the four major serum inflammatory markers for predicting macrolide-refractory *Mycoplasma pneumoniae* pneumonia, Figure S2: Prediction model of identification to macrolide-refractory *Mycoplasma pneumoniae* pneumonia, Supplementary file S1: The list and IRB approval numbers of all the institution related with this study, Table S1: Demographics of the two groups classified by the use of macrolides, Table S2: Pre-existing conditions and their relative distribution according to macrolide response
