# Supplementary material for: Clinical Characteristics of Macrolide-Refractory Mycoplasma pneumoniae Pneumonia in Korean Children: A Multicenter Retrospective Study
Source: J Clin Med. 2022 Jan 8;11(2):306. doi: 10.3390/jcm11020306 (PMC8779611; doi:10.3390/jcm11020306)
Supplement: Supplementary file 1 [file jcm-11-00306-s001.zip › jcm-1499175-supplementary.pdf]

Supplementary material

Supplementary file S1. The list and IRB approval numbers of all the institution related with this study

1. Seoul National University Hospital Approval No.: H-1702-066-831
2. Chungnam National University Hospital Approval No.: CNUH 2017-04-023
3. Chonnam National University Hospital Approval No.: CNUH-2017-094
4. Samsung Changwon Hospital Approval No.: SCMC 2017-02-001-001
5. Inje University Sanggye Paik Hospital Approval No.: SGPAIK 2015-12-019
6. Ulsan University Gangneung Asan Hospital Approval No.: 2017-06-009
7. Inje University Haeundae Paik Hospital Approval No.: 2018-08-008
8. Soonchunhyang University Seoul Hospital Approval No.: SCHUH201-309013001
9. Dankook University Hospital Approval No.: DKUH 2017-04-021
10. Kangbuk Samsung Hospital Approval No.: 2017-02-036
11. University Guro Hospital Approval No.: KUGH17065-001
12. Eulji University Hospital Approval No.: 2017-03-011
13. Korea Cancer Center Hospital Approval No.: K-1703-002-086
14. Asan Medical Center Approval No.: 2017-0566
15. Pusan National University Yangsan Hospital Approval No.: 05-2017-051
16. National Health Insurance Service Ilsan Hospital Approval No.: 2017-02-009-003
17. Daegu Catholic University of Medical center Approval No.: CR-17-035
18. Dongguk University Kyungju Hospital Approval No.: 110757-201703-HR-03-01
19. CHA Bundang Medical Center Approval No.: 2017-06-006
20. The Catholic University of Korea Uijeongbu St. Mary’s Hospital Approval No.: UC17RNMI0021

21. Severance Children's Hospital Approval No.: 4-2017-0166

22. Hallym University Kangnam Sacred Heart Hospital Approval No.:2017-04-007-001

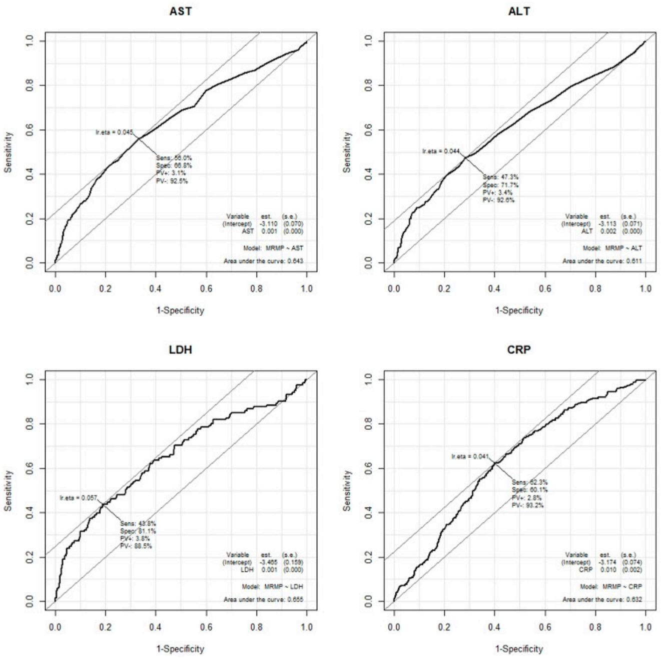

Figure S1: Receiver-operating-curves of the four major serum inflammatory markers for predicting macrolide-refractory *Mycoplasma pneumoniae* pneumonia

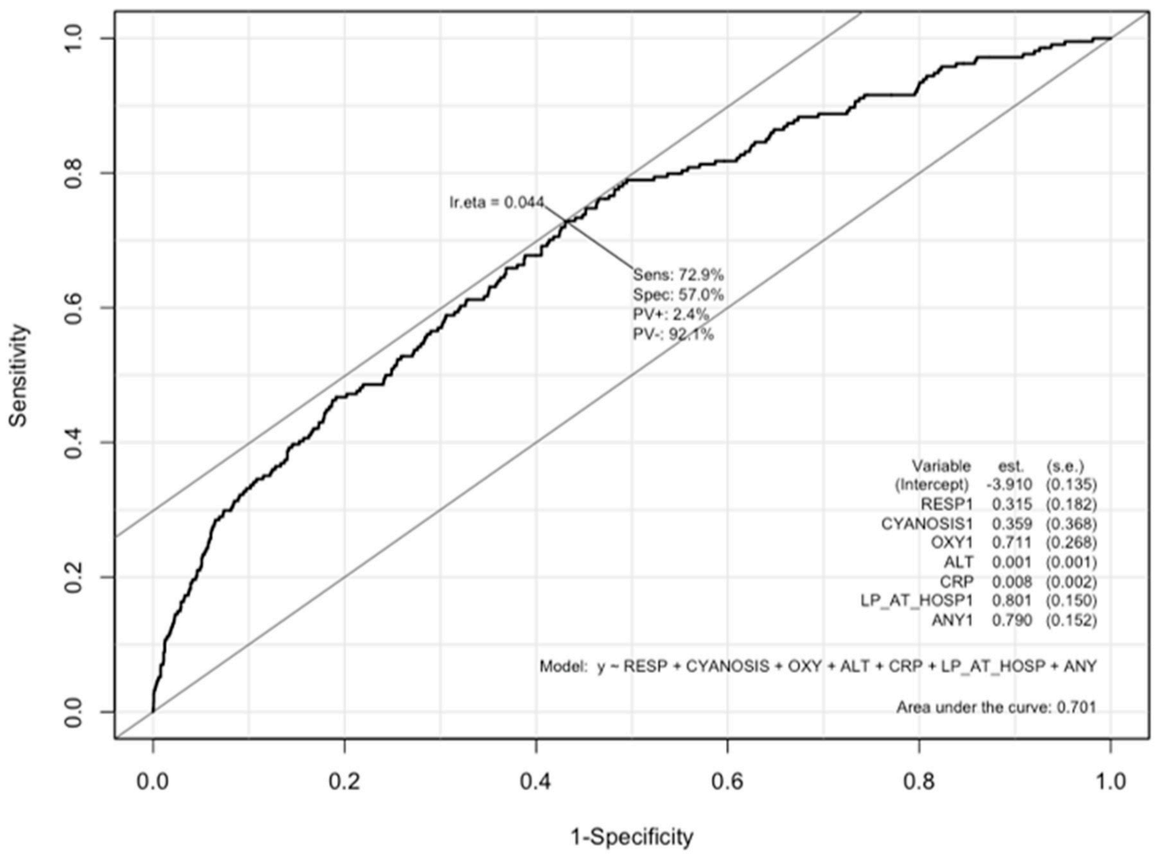

Figure S2: Prediction model of identification to macrolide-refractory *Mycoplasma pneumoniae* pneumonia

Table S1. Demographics of the two groups classified by the use of macrolide

	Patients who did not use macrolide	Children with *Mycoplasma* pneumonia treated with macrolide	*P*-value
Number of the subjects	2175	5294	
Male sex	1094 (50.3)	2596 (49)	0.33
Age (months)	67.5 (41.9)	66.8 (39.4)	0.48

All data are presented as either n (%) or mean (standard deviation [SD])

Table S2. Pre-existing conditions and their relative distribution according to macrolide response

Category	Total population	MRMP group	MLMP group	MSMP group
Number of the subject	5294	240	925	4129
Any pre-existing condition	725/4159 (17.4)	44/216 (20.4)	123/802 (15.3)	558/3141 (17.8)
Allergic disorder	645/4162 (15.5)	38/217 (17.5)	114/802 (14.2)	493/3143 (15.7)
Asthma	334/4811 (6.9)	6/236 (2.5) (236)	42/872 (4.8)	286/3703 (7.7)
Controller in use*	213 (63.8)	1 (0.4)	14 (1.6)	198 (5.3)
Food allergy	141/4993 (2.8)	16/240 (6.7)	28/923 (3)	97/3830 (2.5)
Atopic dermatitis	182/4992 (3.6)	7/240 (2.9)	46/923 (5)	129/3829 (3.4)
Atopic sensitization	261/4623 (5.6)	16/220 (7.2)	50/840 (6)	195/3562 (5.5)
Respiratory disorder				
Bronchopulmonary dysplasia	14/5223 (0.3)	0/240 (0)	1/923 (0.1)	13/4060 (0.3)
Cardiologic disorder				

Congenital heart disease	56/5224 (1.1)	0/240 (0)	3/923 (0.3)	53/4061 (1.3)
Neurologic disorder	47/4996 (0.9)	5/240 (2.1)	6/923 (0.7)	36/3833 (0.9)
Musculoskeletal diseases	15/5341 (0.3)	1/240 (0.4)	0/923 (0)	14/4078 (0.3)
Hemato-oncologic disorder	8/5222 (0.2)	2/239 (0.8)	3/923 (0.3)	3/4060 (0.07)
Immune-deficiency or connective tissue disease	11/5223 (0.2)	0/240 (0)	2/923 (0.2)	9/4060 (0.2)

All data are presented as n (%). *: The proportion of patients using a controller among asthma patients.

For items with missing responses, the number of actual responses was entered in the column on the right.
